# Breeding Sustainable Beef Cows: Reducing Weight and Increasing Productivity

**DOI:** 10.3390/ani12141745

**Published:** 2022-07-07

**Authors:** Warren M. Snelling, R. Mark Thallman, Matthew L. Spangler, Larry A. Kuehn

**Affiliations:** 1 USDA-ARS, US Meat Animal Research Center, Clay Center, NE 68933, USA; mark.thallman@usda.gov (R.M.T.); larry.kuehn@usda.gov (L.A.K.); 2 Animal Science, University of Nebraska-Lincoln, Lincoln, NE 68583, USA; mspangler2@unl.edu

**Keywords:** beef cattle, sustainability, low-pass sequencing, functional variants

## Abstract

**Simple Summary:**

Improving the sustainability of beef cows involves reducing feed costs and enteric methane emissions and increasing calf production while addressing concerns including animal health and welfare and worker safety. Reducing cow weight can favorably impact feed costs and methane emissions. Cumulative weight weaned observed throughout a cow’s productive life directly addresses calf production and indirectly addresses other concerns—cumulative production is higher for cows who wean healthy calves and avoid culling because of reproductive failure, unsoundness, and dangerous behavior. Using functional variant genotypes imputed from the low-coverage whole genome sequence, this examination of cow weight and cumulative weight weaned in a herd of crossbred cattle resulted in additive heritability estimates of 0.57 for cow weight and 0.11 for weight weaned by 8-year-old cows. Corresponding dominance heritability estimates were 0.02 for cow weight and 0.19 for weight weaned. All breeds were represented by cows projected to have high and low cow weights and weight weaned. Heterosis was higher and genomic inbreeding, measured by runs of homozygosity, was lower among high-weight weaned cows. These results suggest selection should be effective in reducing cow weight. Selection to increase weight weaned will be slow but can be hastened with crossbreeding. Especially when pedigree is not available to estimate heterosis, runs of homozygosity may be a useful indicator of heterosis and a predictor of cumulative productivity. Beef cow sustainability can be improved with appropriate crossbreeding and selection, and may be accelerated by incorporating functional variants associated with sustainability-related traits.

**Abstract:**

Programs for sustainable beef production are established, but the specific role of beef cows in these systems is not well defined. This work characterized cows for two traits related to sustainability, cow weight (CW) and cumulative weight weaned (WtW). Cow weight indicates nutrient requirements and enteric methane emissions. Cumulative weight weaned reflects reproductive performance and avoidance of premature culling for characteristics related to animal health, welfare, and worker safety. Both traits were evaluated with random regression models with records from a crossbred population representing 18 breeds that conduct US national cattle evaluations. The genomic REML analyses included additive and dominance components, with relationships among 22,776 animals constructed from genotypes of 181,286 potentially functional variants imputed from a low-pass sequence. Projected to 8 years of age, the additive heritability estimate for CW was 0.57 and 0.11 for WtW. Dominance heritability was 0.02 for CW and 0.19 for WtW. Many variants with significant associations with CW were within previously described quantitative trait loci (QTL) for growth-related production, meat, and carcass traits. Significant additive WtW variants were covered by QTL for traits related to reproduction and structural soundness. All breeds contributed to groups of cows with high and low total genetic values (additive + dominance effects) for both traits. The high WtW cows and cows above the WtW mean but below the CW mean had larger heterosis values and fewer bases in runs of homozygosity. The high additive heritability of CW and dominance effects on WtW indicate that breeding to improve beef cow sustainability should involve selection to reduce CW and mate selection to maintain heterosis and reduce runs of homozygosity.

## 1. Introduction

Various programs have emerged to address the sustainability of beef production [1,2,3,4]. These programs share the three pillars of social, environmental, and economic sustainability [5] and have similar concerns related to natural resources, people and communities, animal health and welfare, food safety and quality, and production efficiency and innovation. Various criteria and practices to address these concerns have been established, but none specifically address the role of the beef cow in sustainable production.

Current evaluations of stayability, the probability of a cow reaching an age of six years [6], may address some aspects of beef cow sustainability. Because reproductive failure is the primary reason for premature culling [7], stayability is usually regarded as a reproductive trait affecting economic sustainability and efficiency. Culling for temperament (dangerous to handle), udder problems, and unsoundness (requiring extra handling or being unable to nurse their growing calves) also address sustainability concerns related to worker safety and animal health and welfare. Stayability can also contribute to environmental sustainability by reducing the fraction of the average cow life cycle spent in the non-productive heifer development phase. While enteric methane emission by cows is unavoidable, selection to increase stayability may reduce methane emissions per breeding cow and per unit of beef produced [8].

The extension of stayability to evaluate cow weight and cumulative cow productivity [9] under a restricted breeding season provides refined tools to breed for sustainability. These evaluations predict the most and least productive cows, and weight indicates feed requirements [10] and methane emissions [11]. Cow weight evaluations [9,12] are also considered in developing a herd suitable for managed grazing prescribed for sustainable beef production [1,2,3,4]. Managed grazing can protect and improve land resources and increase carbon sequestration [13]. Meeting nutrient requirements of each cow in the herd may be simplified if all cows are close to the same weight and on the same breeding—calving—weaning schedule so they have similar nutrient requirements that can be synchronized with nutrient availability from grazed forage [14].

As a step toward developing tools to improve beef cow sustainability, this study examined genetic control of cow weight and cumulative productivity. Using genotypes imputed from low-pass sequence [15,16], specific objectives for a genomic evaluation of cow weight and cumulative productivity in a multibreed herd were to characterize cows that may be the most and least efficient and sustainable, quantify variation attributable to sequence-level genotypes, and identify specific variants associated with weight and productivity.

## 2. Materials and Methods

### 2.1. Data Source

Data for this study were obtained from the ongoing US Meat Animal Research Center (USMARC) Germplasm Evaluation Project (GPE). Animals were raised, phenotypes were observed, and biological samples for genotyping and sequencing were obtained following USMARC standard operating procedures and Federation of Animal Science Societies (FASS) guidelines [17]. Pedigree, birth, and weaning records of all GPE animals were extracted from the USMARC cattle records database, along with breeding assignments and pregnancy test results of GPE females exposed to breeding.

Prior to the fall 2006 breeding season, cows were bred for spring calving only and not culled for reproductive failure until their second consecutive non-pregnant diagnosis. Starting with the fall 2006 breeding, they were bred for spring and fall calving seasons, held over to the next calving season after their first non-pregnant diagnosis, and culled after a second non-pregnant diagnosis. Additional culling was for lameness, udder conformation, temperament, and other issues adversely affecting animal or handler welfare.

Cow weight at pregnancy test (CW) and cumulative weight weaned (WtW) records were obtained on 6211 genotyped females from GPE Cycles VII [18] and VIII [19] and the current 18-breed continuous GPE sampling [20], following procedures developed for random regression analyses of these traits [9]. Briefly, a record for WtW was created each time a female was exposed to breeding, starting with their initial breeding to calve as a two-year-old. The WtW for each breeding was the actual weight of the resulting calf at weaning (zero if a weaned calf did not result from that breeding), plus the sum of previous calves’ weaning weights. Cow weights were recorded when pregnancy was diagnosed via rectal palpation or ultrasound following each breeding season. The age associated with each record for the random regression analyses was the intended age at calving in years, with 0.5-year increments used to accommodate females shifted from the spring (fall) to the fall (spring) breeding season after a non-pregnant diagnosis.

### 2.2. Genotypes

Genotypes from 22,776 GPE animals were used. These included 21,370 animals genotyped with at least one SNP assay (19,576 single assay, 1794 2 to 4 assays) and 2923 animals with sequence variant genotypes imputed from low-pass (~0.5×) whole-genome sequence (WGS; Table 1). The low-coverage sequence was submitted to the Gencove pipeline for imputation with loimpute [21] to a haplotype reference panel constructed from WGS of 946 cattle (598 available from NCBI Sequence Read Archive; 348 GPE sires) [16]. The functional impact of the imputed variants was assessed with snpEff [22] using the Ensembl annotation [23] of the ARS-UCD1.2 assembly of the bovine genome [24]. Genotypes for interesting variants and SNP probed by the BovineHD (Illumina, Inc) and GGP-F250 (Neogen, Inc) assays were extracted from the imputed calls of each individual with a low-pass sequence. Interesting variants included variants in exons of protein-coding genes, which may affect gene function, and variants in untranslated regions (UTR) and non-coding RNA, which may impact gene regulation.

To extend sequence variant genotypes to genotyped GPE animals, low-pass genotypes were combined with SNP array genotypes for pedigree-informed imputation with findhap version 3 [25]. Prior to pedigree imputation, ARS-UCD1.2 positions of array SNP were obtained from the National Animal Genome Research Program (NAGRP) data repository [26]. Array genotypes, expressed as 0, 1, or 2 copies of allele B, were translated to 0, 1, or 2 copies of the alternate allele with the aid of the “.REF” files archived in [26], which list nucleotides associated with the A and B alleles. 

Low-pass calls were required to have a genotype probability greater than 0.95. A 0.95 call rate filter by animal and variant was applied to the set of interesting variants and array SNP extracted from low-pass genotypes. The same call rate filter was applied to each SNP array used to genotype GPE animals. Pedigree imputation with findhap was first used to impute animals with lower density array genotypes to the set of SNP probed by the BovineHD and GGP-F250 arrays. Low-pass genotypes for the interesting sequence variants were then added to impute from BovineHD + GGP-F250 up to sequence variants. In a test of accuracy, all interesting sequence variants were included in the first round of imputation, but the sequence variant genotypes of the 96 2017-born animals with low-pass were excluded. For each variant, correlations (r) between the low-pass calls of those animals and genotypes imputed with findhap were computed. The final round of pedigree imputation included all low-pass genotypes for variants with r > 0.8 or r undefined due to lack of variation in the test animals.

After imputation, genomic relationship matrices (GRM) were constructed. Intergenic and intronic SNP from the arrays were removed, so the GRM represented variants expected to have functional consequences. Variants in the GRM had minor allele frequencies greater than 0.005, and close (within 50 kbp), nearly redundant (r > 0.98) variants were removed using the snpgdsLDpruning function of SNPRelate [27]. After filtering, genotypes for 181,286 variants were used to construct two GRMs, an additive GRM (G) following [28] and a dominance GRM (D) built according to [29]. 

Random regression analyses of WtW and CW were similar to the previous analyses of GPE cows [9], except that GRM was used instead of pedigree relationships, and only univariate analyses were conducted. Fixed effects included birth year-season-composition opportunity groups, where GPE females were assigned to mating groups by the maximum composition of any one breed (50% to <75%; 75% to <87.5%; ≥87.5%), and intended age at calving, in half-year increments to accommodate shifts from spring (fall) to fall (spring) calving seasons. A term for cumulative calf sex (males weaned—females weaned) was included in the WtW analysis to account for the sex difference in calf weaning weight. Random animal effects were modeled with additive relationships described by G and dominance relationships described by D. Variance components for each trait were estimated with restricted maximum likelihood algorithms implemented in WOMBAT [30]. Additive and dominance animal effects were projected to age 8 years. The 8-year-old projections were used to characterize cows by genetic merit for weight and productivity and to solve the effects of individual variants on cow weight and productivity. Observed cows were split into halves above and below the mean total merit (additive + dominance effects) for each trait and into quadrants by means of both traits. Breed composition and expected retained heterozygosity, based on pedigree records and expressed as a fraction of F_1_ heterozygosity [31], were summarized for each half and quadrant. Composite breed contributions were split into their component breeds: Brangus (3/8 Brahman, 5/8 Angus), Santa Gertrudis (3/8 Brahman, 5/8 Shorthorn), Beefmaster (1/2 Brahman, 1/4 Hereford, ¼ Shorthorn), MARC II (1/4 Angus, ¼ Hereford, ¼ Gelbvieh, ¼ Simmental), MARC III (1/4 Angus, ¼ Hereford, ¼ Red Poll, ¼ Pinzgauer) and ChiAngus (0.8 Angus, 0.2 Chianinia). Angus and Red Angus were considered the same breed to compute expected retained heterozygosity. Additive variant effects were solved by $\hat{\alpha }=M^{\prime }{\left[MM^{\prime }\right]}^{-1}\hat{u_{a}}\;$[32], where $\hat{\alpha }$ is a vector of additive variant effects, M a matrix of additive genotypes and $\hat{u_{a}}$ a vector of predicted additive animal effects. Similarly, dominance effects were solved by $\hat{d}=H^{\prime }{\left[HH^{\prime }\right]}^{-1}\hat{u_{d}}$**,** where $\hat{d}$ is a vector of dominance variant effects, **H** a matrix of heterozygosity coefficients, and $\hat{u_{d}}$ a vector of predicted dominance animal effects. Z-scores were computed for each vector of variant effects, and the standard error of the z-scores from 5000 random permutations of each animal effect vector [33] was used to approximate *p* of each variant. Significant variants were identified by Bonferroni-corrected *p* < 0.05. Variants excluded from **M** and **H** because of redundancy were assumed to have the same significance as the **M** and **H** variants with which they were redundant. The CattleQTL database from AnimalQTLdb [34] was examined to determine published quantitative trait loci (QTL) overlapping significant variants.

### 2.3. Genomic Heterosis

The genotypes used to construct GRM were also used to compute genomic measures that may reflect heterosis. These included genomic inbreeding (F_g_) coefficients taken from the diagonal of the additive GRM; heterozygosity (gHet), the fraction of each animal’s heterozygous genotype calls, bases within heterozygous-rich regions (HRR) [35], and bases under runs of homozygosity (ROH). The R package detectRUNS [36] was used to detect both HRR and ROH in each animal. The consecutive method was used with a minimum ROH length of 1 Mb following [37], and a minimum HRR length of 100 Kb. Projected dominance and total effects on CW and WtW were regressed on retained heterozygosity and each genomic measure, and the metrics were summarized for cow weight and cumulative productivity groups.

## 3. Results

**Figure 1:**
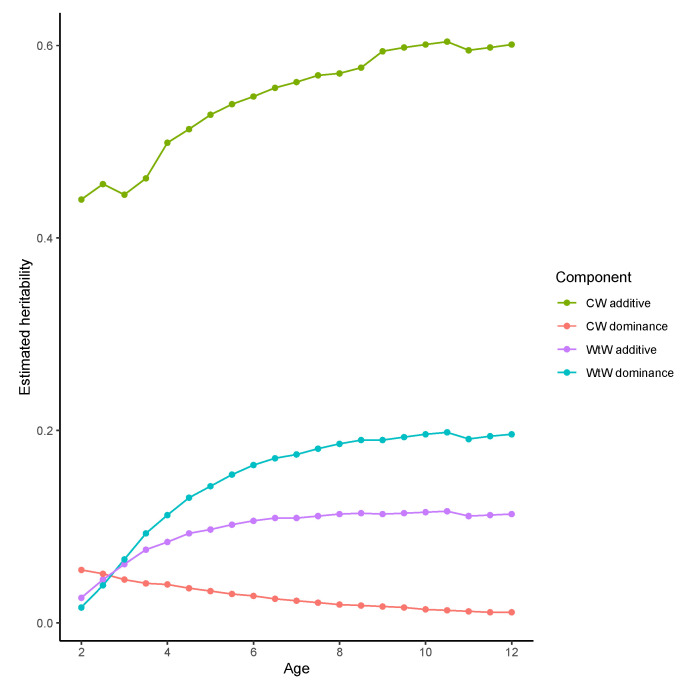
Projected additive and dominance heritability estimates from random regression analysis of cow weight (CW) and cumulative weight weaned (WtW).

Observed weights of 8-year-old cows averaged 609 kg, with a 663 kg range, from 284 to 948 kg (Table 2). Projected to age 8 years, additive effects of observed cows explained more than half of the observed difference, while dominance effects accounted for less than 5% of the total genomic variance. The observed 1956 kg range of cumulative weight weaned by 8-year-olds includes a cow that tested pregnant after 6 of her 7 breeding seasons but subsequently failed to calve or lost calves within a day after birth. Genomic animal effects also account for more than half the observed difference in WtW by 8-year-olds, although the difference due to dominance is greater than the additive difference. Projected across the range of observed cow age, estimated additive effects on cow weight are consistently strong, accounting for about 50% of phenotypic variation (Figure 1). Dominance effects on cow weight were small at all ages. There was little genomic influence (additive + dominance) on calf weight weaned by first-calf heifers. Still, the genomic influence on cumulative weight weaned increased with age, explaining about 30% of the variation in weight weaned by 8-year-old cows. After age 3, dominance variance exceeded additive and was almost twice the additive variance by age 8.

### 3.1. Breed Contributions and Heterosis

**Figure 2:**
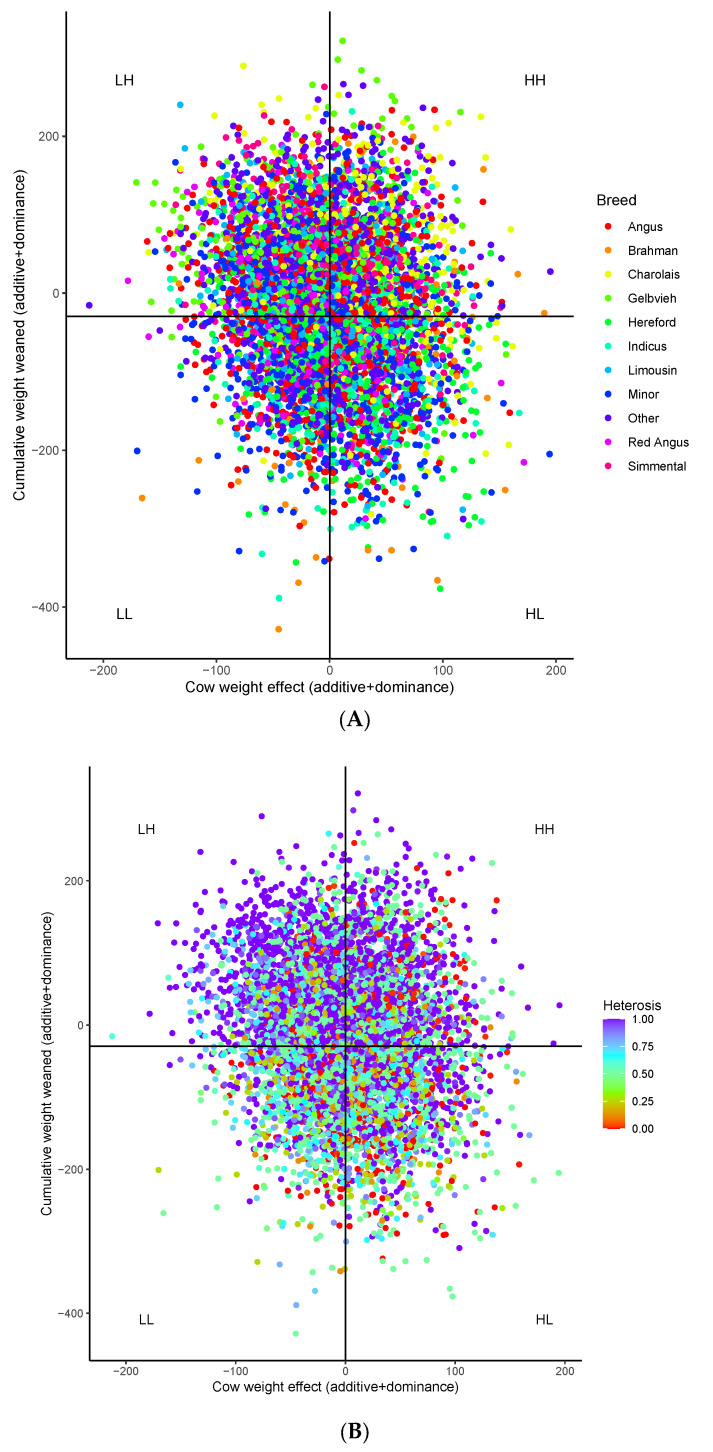
Total genetic effects (additive + dominance) of individual cows projected to eight-year-old cumulative weight weaned (WtW) and cow weight (CW). Each dot represents a single cow. The mean cow weight effect is shown by the vertical line, and the mean cumulative weight weaned effect by the horizontal line. Quadrants below and above the means for each trait are labeled: low CW/low WtW (LL), low CW/high WtW (LH), high CW/high WtW (HH), and high CW/low WtW (HL). Colors reflect breed composition (**A**) and level of heterosis (**B**). Individual breeds in A include cows who are at least 50% of one of the major breeds. Indicus includes cows that are at least 50% of one of the *Bos indicus*-influenced composites (Brangus, Beefmaster, Santa Gertudis). Minor combines cows that are at least 50% of a minor breed (Braunvieh, ChiAngus, Maine-Anjou, Salers, Shorthorn, South Devon, and Tarentaise), and Other are cows less than 50% of any single breed.

All breeds contributed to groups of cows above and below means total merit for both 8-year-old weight and cumulative weight weaned, as well as to quadrants defined by means of both traits (Figure 2A). There were differences, however, in the composition of each group. Mean Angus, Braunvieh, Gelbvieh, Limousin, and Red Angus composition of the low CW group was greater than the high CW group. In contrast, Brahman, Charolais, Maine-Anjou, Salers, and Simmental composition was higher in high CW cows (Supplementary Materials). Breeds with greater contributions to the high WtW group included Charolais, Gelbvieh, Simmental, and Tarentaise, while Angus, Brahman, Braunvieh, Chianinia, Hereford, Maine-Anjou, Red Angus, and Shorthorn had increased contributions to the low WtW cows. By CW/WtW quadrants, Angus, Hereford, Braunvieh, Red Angus, and Shorthorn were overrepresented in the low CW/low WtW group; Angus, Simmental, Gelbvieh, Limousin, and Tarentaise in low CW/high WtW; Hereford, Brahman, Shorthorn, Maine-Anjou and Salers in high CW/low WtW; and Charolais and Simmental in the high CW/high WtW group. 

Retained heterozygosity was higher for low-weight cows and cows with high cumulative weight weaned. While the full range of retained heterozygosity, from purebred to F_1_, was observed in all groups, the mean retained heterozygosity of the high-weight weaned cows was greater than low weight weaned cows (Figure 2B; Supplementary Materials). F_1_ cows were most common in the high-weight weaned groups, with about 50% of the high-weight weaned cows being F_1,_ but only 36% of low-weight weaned cows were F_1_. About 40% of both the low and high cow weight groups were F_1_.

### 3.2. Genomic Heterosis

For WtW, the trait with a substantial dominance effect, regression R^2^ (Table 3) indicated that bases under ROH were the genomic measure that explained the most variation in dominance effects and total additive and dominance effects. Bases in ROH accounted for 11.6% of the variation in total WtW, approximately twice the 5.6% explained by pedigree heterosis. Heterozygosity accounted for the most variation in dominance effects on CW, but less than 0.5% of total CW variation due to the lack of influence of dominance on CW. Variation explained by bases in HRR was generally small. Cow groups with the highest pedigree heterosis (High WtW, low CW/high WtW; Table 4) had lower than average genomic inbreeding, fewer bases in heterozygosity-rich regions, and fewer bases in runs of homozygosity. Above-average WtW cows had 36 fewer Mb covered by ROH than cows with below-average WtW, and there was a 48 Mb difference between the most and least efficient quadrants CW/WtW quadrants. 

The strongest agreement between pedigree heterosis and genomic measures was with ROH (r = −0.648). Other correlations were ±0.20 or weaker. The strongest correlation among genomic measures was between heterozygosity and genomic inbreeding (r = 0.723); other correlations were weaker than ±0.25.

### 3.3. Genomic Variant Effects

**Figure 3:**
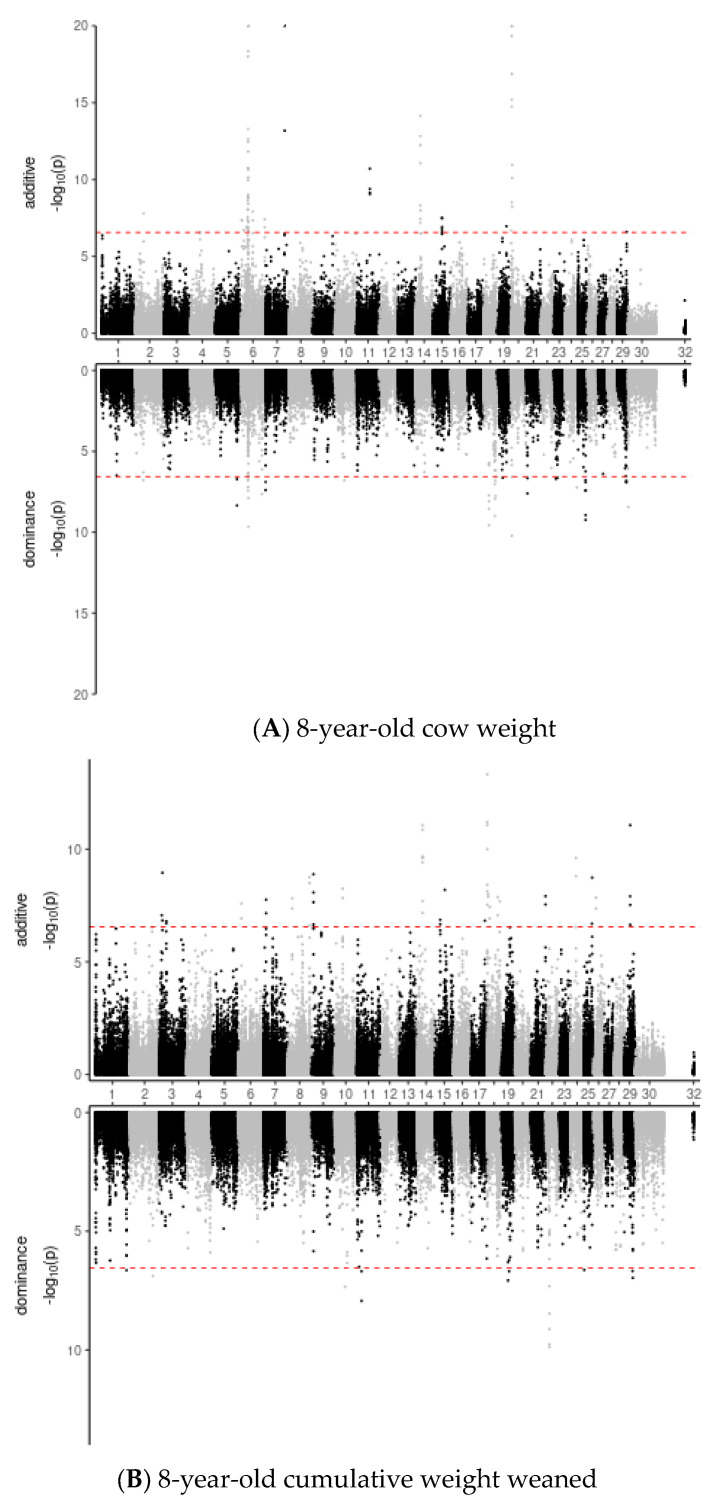
Miami plots depicting significance of variants affecting projected 8-year-old cow weight (**A**) and cumulative weight weaned (**B**). Additive effects are above the axis, dominance effects are below. Chromosomes are autosomes 1 to 29, X (30), and the pseudoautosomal region of X (32).

One hundred ninety-three variants with significant effects (Bonferroni-corrected *p* > 0.05) on at least one trait and component were identified (Figure 3; Supplementary Table S2). Predictions of variant effects [22] using Ensembl annotation [23] indicated 107 genes could be affected by these variants (Table 5). The identified variants included 38 that would alter the amino acid sequence of coded proteins and 3 expected to cause more severe changes to the protein. The 159 remaining variants may affect phenotype without altering protein sequences. These occur in untranslated regions (UTR) of protein-coding genes or exons of non-coding features and could have regulatory effects on coded proteins. Synonymous SNP, which does not alter the amino acid sequence, might still affect gene expression and function [38,39].

The variants significant for any trait and component were overlapped by 986 published QTL [34]. Overall, production traits and meat and carcass traits were the predominant categories (Table 6). Almost 56% of the QTL covering a significant variant were in one of those two categories, and more than 67% of QTL covering variants associated with the additive component of cow weight were in those two categories. Less than 5% of the QTL containing additive CW variants were for reproductive traits, but over 15% of the QTL covering additive WtW variants were for reproductive traits. Milk-related QTL was most prominent for variants associated with the dominance component of both traits. Health and exterior QTL, which include behavior, structural soundness, and other convenience traits, were more prominent among QTL, covering variants associated with additive WtW effects and dominance effects on both CW and WtW.

## 4. Discussion

The most sustainable cows observed in this study are the low cow weight and high cumulative weight weaned cows. These cows should have lower resource requirements and lower enteric methane emissions than heavier herd mates, while avoiding reproductive failure and other reasons for premature culling to wean more total calf weight. These traits also contribute to economic sustainability. Cumulative weight weaned captures reproductive traits which have the greatest emphasis in economic selection indexes for cattle production [40,41,42], and mature cow weight influences feed costs and have a negative impact on selection indexes [42,43].

Although there were some differences in breed composition of these and less sustainable cows, all breeds used in GPE were represented by the high and lower sustainability groups. Reflecting a large dominance contribution to cumulative weight weaned, perhaps the most noticeable features of the high sustainability cows were their high levels of expected retained heterozygosity, with a mean of 0.80 for the most sustainable cows compared to 0.64 for the least sustainable heavy cows with low productivity, and low levels of bases in runs of homozygosity, with the most sustainable cows having 63% fewer bases under ROH than the least sustainable. 

Further results of this study indicate selection and mating decisions can contribute to improving sustainability. The high additive heritability of cow weight indicates selection will effectively reduce cow weight. Due to lower additive heritability, selection to increase cumulative weight weaned will be slow. Still, it can be accelerated by breeding systems to address heterosis [44,45] and reduce bases in runs of homozygosity. A composite system, where sires and dams share the same mixed-breed composition, may be somewhat more sustainable than rotational crossbreeding, mating purebred sires to crossbred dams. Both approaches can produce breeding females with similar levels of heterosis. Still, rotational crossbreeding requires bulls sourced from populations of purebred females with no heterosis, and the multiple breeding herds needed to implement rotational crossbreeding fully are less amenable to managed grazing. When a split cow herd can be managed, sustainability might be improved by mating a portion of the herd to high-growth terminal sires to increase weight weaned.

Possible functional variants were associated with CW and WtW. While further examination of these variants is needed, many coincide with established QTL: CW-associated variants with growth and weight-related QTL, and WtW with reproduction, soundness, and convenience traits influencing animal welfare and sustainability. The additive variants may add accuracy to selection, and dominance variants could be used to sort heifer calves into market and breeding groups. Examining these variants and traits in other populations is needed. In particular, heterosis and dominance effects might be exaggerated by the 18-breed GPE design, where chances of producing an F_1_ female sired by a breed outside her pedigree are greater than in composite and crossbred herds that may use three or four breeds. 

Genomic inbreeding and heterozygosity were positively correlated, opposite to the expectation that inbreeding reflects homozygosity. Due to the influence of allele frequency on additive G [28], heterozygous genotypes of low-frequency variants have a positive contribution to the genomic inbreeding taken from the diagonal of G. The correlation of ROH with expected retained heterozygosity and association with WtW suggest ROH may be the most useful of the genomic measures of heterosis. A similar but positive correlation was found between ROH and pedigree inbreeding [46], and ROH has been associated with inbreeding depression in cattle [46,47]. These results associating ROH with heterosis concur with the idea that heterosis is the recovery of inbreeding depression resulting from breed formation [31]. Pedigree-based inbreeding and retained heterozygosity coefficients, however, are expressed on different scales. ROH will allow a single term to account for both inbreeding and heterosis and can be measured on cattle genotyped with a moderately dense assay or low-coverage sequence, with or without pedigree and breed composition records needed to compute expected retained heterozygosity values.

**Figure 4:**
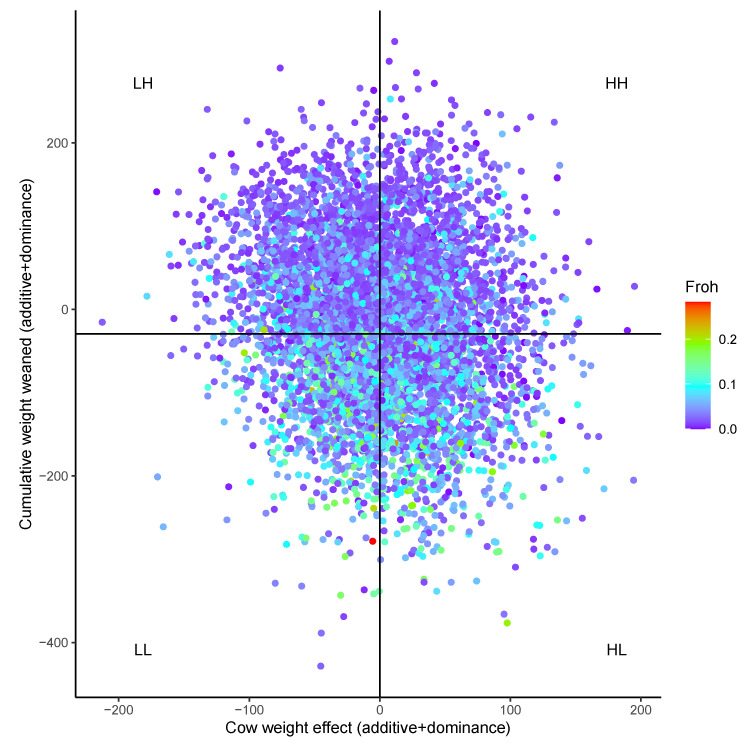
Total genetic effects (additive + dominance) of individual cows projected to eight-year-old cumulative weight weaned (WtW) and cow weight (CW). Each dot represents a single cow. The mean cow weight effect is shown by the vertical line, and mean cumulative weight weaned effect by the horizontal line. Quadrants below and above the means for each trait are labeled: low CW/low WtW (LL), low CW/high WtW (LH), high CW/high WtW (HH), and high CW/low WtW (HL). Colors reflect inbreeding measured by bases under runs of homozygosity (F_roh_).

Further examination of ROH effects and manipulation of ROH is needed. While retained heterozygosity and ROH have a moderate negative correlation, so that ROH is expected to decrease as heterosis increases, these data contain cows with zero retained heterozygosity and relatively few bases covered by ROH. The relationship between WtW and ROH appears stronger than between WtW and retained heterozygosity. While the depiction of retained heterozygosity values relative to cow weight and cumulative productivity (Figure 2B) shows most high WtW cows have high heterosis, some zero retained heterozygosity purebred cows are evident. No high ROH (corresponding to low retained heterozygosity) cows are evident in a similar plot of ROH (Figure 4), indicating that low ROH in purebreds, composites, and crossbreeding systems with a manageable number of breeds is possible.

## 5. Conclusions

The CW and WtW traits examined in this study are based on easily measured, routine records that can immediately be implemented in current cattle evaluation schemes. Various measures of forage intake and methane emissions have been developed [48,49,50] that may more directly address those components of sustainability. The individual measurements of grazing intake and methane emissions are generally expensive and may be too labor-intensive to obtain records on enough cows for meaningful evaluations based on these traits alone. Until suitable databases can be developed or useful indicator traits identified, selection to reduce cow weight in crossbreeding systems to minimize ROH and increase lifetime production represents the most tangible strategy for breeding sustainable cows.

## Figures and Tables

**Table 1:** Genotypes available from the Germplasm Evaluation project population.

Platform	SNP	Sires	Dams	Nonparents	Total
SNP50 v1 ^1^	54,115	1245	1064	2466	4775
SNP50 v2 ^1^	54,042	90	956	4140	5186
BovineHD ^1^	774,990	921	467	162	1550
GGP ^2^-F250 ^3^	206,629	1435	561	371	2367
GGP v1 ^2^	76,570	0	0	517	517
GGP v2 ^2^	19,640	0	0	172	172
GGP v3 ^2^	25,969	0	816	2635	3451
GGP v4 ^2^	29,704	0	154	789	943
GGP 50 K ^2^	44,739	0	1210	2612	3822
GGP 100 K ^2^	93,843	1	177	971	1149
All arrays	911,640	1886	4917	14,567	21,370
Low-pass	59,204,180	412	2375	136	2923
Low-pass + arrays	59,280,638	2013	6088	14,675	22,776

^1^ Illumina Inc., San Diego, CA, USA; ^2^ GeneSeek Genomic Profiler, Neogen Inc., Lincoln, NE, USA; ^3^ functional variant assay.

**Table 2:** Summary of observed 8-year-old cow weight (CW; kg) and cumulative weight weaned (WtW; kg), and predicted genomic additive and dominance effects.

Trait		Minimum	Maximum	Mean	SD
CW	observed	284.4	947.8	609.1	82.5
	additive	−197.5	194.3	0.4	50.8
	dominance	−15.1	20.3	−0.5	3.7
WtW	observed	0.0	1955.6	1286.3	253.8
	additive	−297.6	163.2	−3.4	55.3
	dominance	−267.6	289.2	−10.0	65.4

**Table 3:** Percentage of variation (R^2^ × 100) in projected dominance and total (additive + dominance) effects on cow weight (CW) and cumulative weight weaned (WtW) explained by pedigree-based retained heterozygosity (pHet) and genomic indicators of heterosis ^1^.

	Dominance		Total	
	CW	WtW	CW	WtW
pHet	6.53	13.14	1.52	5.61
gHet	17.63	11.08	0.46	0.03
Fg	6.95	1.22	1.02	3.40
HRR	4.12	0.90	0.57	0.38
ROH	12.60	21.26	0.01	11.65

^1^ Genomic heterozygosity (gHet) = heterozygous genotypes/total genotypes; genomic inbreeding (Fg) = diagonal of G − 1; bases in heterozygosity-rich regions (HRR); bases in runs of homozygosity (ROH).

**Table 4:** Means (SE) of pedigree-based retained heterozygosity (pHet) and genomic indicators of heterosis ^1^ for cows grouped by projected effects on cow weight (CW) and cumulative weight weaned (WtW).

Cow Group ^2^		Genomic Indicators				
	pHet	gHet	Fg	HRR	ROH	*n*
All	0.711 (0.004)	0.349 (0.0003)	0.006 (0.002)	9072758	106867400	6211
				(26512)	(1193523)	
Low CW	0.742 (0.005)	0.347 (0.0005)	−0.006 (0.002)	9072758	106584500	3114
				(34543)	(1662454)	
High CW	0.680 (0.006)	0.350 (0.0005)	0.018 (0.003)	9182985	107151900	3097
				(40165)	(1713475)	
Low WtW	0.700 (0.006)	0.353 (0.0006)	0.036 (0.003)	9389333	117693600	3065
				(40696)	(1820098)	
High WtW	0.769 (0.005)	0.349 (0.0004)	−0.011 (0.002)	8961127	81655680	3219
				(35280)	(1220320)	
LL	0.662 (0.009)	0.347 (0.0008)	0.016 (0.004)	8992696	137128100	1356
				(53574)	(2934484)	
LH	0.803 (0.006)	0.347 (0.0005)	−0.022 (0.002)	8940328	83025260	1758
				(45131)	(1681042)	
HH	0.727 (0.009)	0.352 (0.0007)	0.003 (0.003)	8986154	80007690	1461
				(55628)	(1770986)	
HL	0.637 (0.008)	0.349 (0.0009)	0.031 (0.004)	9358762	131392600	1636
				(57228)	(2694706)	

^1^ Genomic heterozygosity (gHet) = heterozygous genotypes/total genotypes; genomic inbreeding (F_g_) = diagonal of G − 1; bases in heterozygosity-rich regions (HRR); bases in runs of homozygosity (ROH). ^2^ Low CW—below mean CW effect; High CW—above mean CW effect; Low WtW—below mean WtW effect; High WtW—above mean WtW effect; LL—below mean CW and below mean WtW; LH—below mean CW and above mean WtW; HH—above mean CW and above mean WtW; HL—above mean CW and below mean WtW.

**Table 5:** Number of variants, affected genes, and functional impact of variants associated ^1^ with additive and dominance effects on 8-year-old cow weight (CW) and cumulative weight weaned (WtW).

			CW		WtW	
		All	Additive	Dominance	Additive	Dominance
Variants Genes		366	139	68	120	46
		120	46	27	37	15
**Functional annotation**						
**Impact**	**Annotation**					
HIGH	splice donor; intron	1	1			
HIGH	start_lost	1			1	
HIGH	stop_gained	1	1			
MODERATE	nonsynonymous	67	31	7	26	5
MODIFIER	3′ UTR	111	45	31	20	16
MODIFIER	5′ UTR	33	17	4	10	3
MODIFIER	noncoding exon	16	3		9	4
LOW	5′ UTR; premature start codon gain	1	1	1		
LOW	splice region; intron	11	3	2	4	2
LOW	splice region; synonymous	2			1	1
LOW	synonymous	131	39	23	54	17

^1^ Bonferroni-corrected *p* (*p*_c_) < 0.05 or excluded from analysis because of redundancy with variant with *p*_c_ < 0.05.

**Table 6:** Number (%) of QTL containing variants associated ^1^ with additive and dominance effects on 8-year-old cow weight (CW) and cumulative weight weaned (WtW).

		CW		WtW	
Trait Category	All	Additive	Dominance	Additive	Dominance
Exterior	79 (8.0)	29 (5.4)	36 (11.4)	33 (11.0)	15 (10.1)
Health	91 (9.2)	35 (6.5)	44 (13.9)	36 (12.0)	17 (11.4)
Meat and Carcass	238 (24.1)	150 (27.9)	60 (18.9)	67 (22.3)	28 (18.8)
Milk	171 (17.3)	92 (17.1)	78 (24.6)	58 (19.3)	38 (25.5)
Production	313 (31.7)	211 (39.3)	61 (19.2)	60 (20.0)	33 (22.1)
Reproduction	94 (9.5)	20 (3.7)	38 (12.0)	46 (15.3)	18 (12.1)
Total	986 (100)	537 (100)	317 (100)	300 (100)	149 (100)

^1^ Bonferroni-corrected *p* (*p*_c_) <0.05 or excluded from analysis because of redundancy with a variant with *p*_c_ < 0.05.

## Data Availability

Data generated by this project, including summaries of animal effects and variant effect estimates, are available from https://doi.org/10.5281/zenodo.6595838.

## Supplementary Materials

The following are available online at https://www.mdpi.com/article/10.3390/ani12141745/s1, Table S1: Mean breed composition and expected retained heterozygosity metrics of cows grouped by predicted effects on cow weight and cumulative weight weaned, Table S2: Effects of potentially functional variants associated with cow weight and cumulative weight weaned.
